# Enhancing yield and quality: research and practice of agro-forest waste for *Lentinus edodes* (shiitake mushroom) cultivation

**DOI:** 10.3389/fnut.2025.1538039

**Published:** 2025-03-19

**Authors:** Hongyan Xu, Guocai Han, Yanyan Li, Qing Meng, Yang Zhang, Yayi Wang, Songling Li

**Affiliations:** ^1^Academy of Agriculture and Forestry Sciences, Qinghai University, Xining, Qinghai, China; ^2^Laboratory for Research and Utilization of Qinghai Tibet Plateau Germplasm Resources, Xining, China; ^3^Qinghai Water Conservancy and Hydropower Survey and Design Institute Co., Ltd., Xining, China; ^4^College of Eco-Environmental Engineering, Qinghai University, Xining, China

**Keywords:** agro-forest waste, shiitake mushroom, alternative substrates, nutritional value, principal component analysis

## Abstract

**Introduction:**

Current research primarily focuses on exploring and developing innovative substrates for cultivating *Lentinus edodes*, aiming to address substrate shortages and the continuous rise in production costs.

**Methods:**

This study uses *Quercus* (oak) sawdust (OS) as a control to evaluate the potential of *Korshinsk peashrub* (KP), *Hippophae rhamnoides* (seabuckthorn) pruning (HRP), and *Lycium barbarum* (goji) pruning (LBP) in the cultivation of *L. edodes* by measuring parameters such as mycelium growth, yield, protein, fat, fiber, amino acids, soluble sugars, and organic acids. Furthermore, principal component analysis and official script function analysis were used to investigate the influence of the matrix formula ratio on the nutritional values of shiitake mushrooms.

**Results:**

Results showed that the average duration to complete stages 2 and 3 of mycelial growth on 10% KP substrates significantly decreased compared to the OS group by 11.0 and 10.7 days, respectively. The weight of mushrooms produced from all agro-forest waste substrates was significantly lower than that of the control group, decreasing by 18.96 to 53.88%. The average mushroom weight for KP groups ranged from 235.37 g/kg to 252.27 g/kg, which was statistically higher than that of the LBP treatments, which ranged from 143.56 g/kg to 165.96 g/kg. However, the protein content in the 10% LBP and 10% HRP groups was significantly higher than that of the control, with increases of 4.69 and 12.89%, respectively, and fiber content also improved, increasing by 3.98 to 12.59%. Furthermore, the content of sweet-tasting amino acids in the 10% KP and 20% KP groups significantly increased compared to the OS group (by 34.86 and 144.92%, respectively). The 30% LBP and 10% KP-10% LBP-10% HRP groups exhibited higher glucose values compared to the OS (increased by 118.71 and 72.26%, respectively). Interestingly, the addition of LBP and KP to the substrates promoted the synthesis of acetic acid in shiitake mushrooms, while this organic acid was not detected in the OS. In summary, shiitake mushrooms cultured in 20% KP, 10% LBP, or 10% KP-10% LBP-10% HRP demonstrated significantly better overall performance.

**Discussion:**

This approach not only reduces operational costs by at least 1,680 RMB but also contributes to environmental sustainability by diverting 2,400 kg of agro-forest waste from landfills. Consequently, the utilization of agroforestry waste serves as an effective strategy not only for environmental protection and cost reduction during mushroom production but also for enhancing the nutritional value of shiitake mushrooms. This, in turn, helps combat malnutrition and contributes to national food security.

## 1 Introduction

Mushrooms are considered healthy foods due to their low-calorie content and various beneficial components, such as polysaccharides, phenolics, and triterpenes (1). *Lentinus edodes* is one of the most popular and commercially favored edible mushroom species (2, 3). China accounts for approximately 72% of global mushroom production, solidifying its position as the world’s leading mushroom producer. Notably, the cultivation of *L. edodes* (shiitake mushroom) represents approximately 25% of China’s total mushroom output (4). In 2022, China produced approximately 12.96 million tons of *L. edodes*, representing 98.3% of the total global production of shiitake mushrooms (5). In the wild, *L. edodes* are typically found growing on hardwood trees, particularly those from the *Fagaceae* family, including oak, beech, and chestnut (6). On an industrial scale, the conventional substrate for cultivating *L. edodes* typically consists of approximately 80% hardwood sawdust, primarily from oak, and 20% starch-rich additives, such as wheat bran or rice bran. In China, sawdust has emerged as one of the main substrates for cultivating *L. edodes* due to its excellent aeration, water retention, nutrient supply, and easy availability; its proportion usually accounts for approximately 50% of bagged substrates (7). With the rapid expansion of *L. edodes* cultivation areas, substrate shortages and rising production costs have become significant challenges for the industry. Numerous research and industry efforts are currently underway to address these issues.

The mycelium of *L. edodes* can secrete lignin-degrading enzymes, enabling it to produce fruiting bodies on wood or similar artificial materials. Various wood products and agricultural byproducts serve as substrates for the commercial production of *L. edodes* (8). Currently, *L. edodes* can be cultivated on a diverse range of lignocellulosic substrates, such as coffee grounds, sugarcane bagasse, grain straw, vineyard prunings, and sorghum stubble (9–16). These alternative substrates not only enhance the growth of *L. edodes* but also contribute to resource recycling and minimize the environmental impact of crop waste. Therefore, the research to explore low-cost and locally adapted alternative substrates for *L. edodes* will continue as the scale of *its* cultivation expands.

Cash tree production has rapidly increased in China over the last decade, making it one of the largest fruit producers in the world (17, 18). Due to its excellent salt tolerance, drought resistance, fast growth, and ability to produce fruit in the first year of planting, *Lycium* (Goji) is widely used for improving saline land and supporting rural economic development. As a result, the area devoted to *Lycium* cultivation has expanded in northern China over the last few decades (19). The estimated production areas of *Lycium* cover approximately 880 million m^2^, extending across the entire northwest to central China, including Xinjiang, Ningxia, Gansu, Qinghai, Shanxi, Inner Mongolia, and Hubei (20). As an ecological protection plant, the annual yield of *Caragana korshinsk* is estimated at approximately 550,000 tons and is widely distributed in northeastern, northwestern, and northern China, representing a significant lignocellulosic biomass resource (21, 22). Moreover, as one of the most effective species for soil and water conservation, the total area of *Hippophae rhamnoides* (seabuckthorn) in China is approximately 2.7 million m^2^, accounting for more than 90% of the world’s growth area (23). Notably, these shrub plants exhibit remarkable branching and regenerative abilities, requiring regular pruning every 4–5 years. The majority of *Korshinsk peashrub* (KP), *Hippophae rhamnoides* pruning (HRP), and *Lycium barbarum* pruning (LBP) are not handled properly, representing a substantial lignocellulosic biomass resource. These materials are often either burned or discarded along orchard edges. However, their high content of lignocellulose leads to poor feed quality, while incineration is not in line with the existing policies in China, making the stroma utilization of pruning waste a problem.

According to early studies, these three types of agro-forest wastes have the potential for mushroom cultivation. For instance, KP has been shown to accelerate the growth rate of *Pleurotus tuoliensis* (24) and enhance the fruit body yield of *Pleurotus eryngii* (22). When used at a proportion of 78% (by volume), KP improved the protein, crude fiber, crude fatty acid, polysaccharide, and total sugar contents of *L. edodes* (21). Additionally, the use of 20% HRP (by volume) significantly improved the mycelial biomass and total cellulose enzyme activity of *L. edodes* (25). Similarly, LBP also has the potential to be used for the cultivation of *L. edodes* due to its wide variety of phytonutrients, including proteins, minerals, vitamins, and functional components (26–29).

However, the use of these pruning waste materials for mushroom cultivation has not yet been investigated. Therefore, this study represents the first attempt to explore the potential of locally available KP, HRP, and LBP, either individually or in various combinations, for growing the shiitake strain “Qihe#1.” Furthermore, it examines alternative local substrate formulations to replace the conventional oak sawdust substrate used in *L. edodes* production.

## 2 Materials and methods

### 2.1 Tested substrates

The experiment was conducted at the Center for Mushroom Studies in Qinghai Province, under the Qinghai Academy of Agricultural and Forestry Sciences, China (E101°49′17″, N36°34′03″). In this experiment, 10 treatments (substrate formulations) were established, each repeated 12 times (120 bags of 1,000 g fresh weight) using a completely randomized design method. The 10 tested substrate formulations consisted of a mixture of OS (oak sawdust), *Korshinsk peashrub* (KP), *Hippophae rhamnoides* pruning (HRP), and *Lycium barbarum* pruning (LBP) in varying ratios (Table 1). It is important to note that the maximum amount of alternative sawdust was limited to 30%, based on findings from our pilot study (unpublished). Subsequently, oak sawdust was added to the mixture to ensure that the total proportion of sawdust reached 80%. Similarly, 180 g of wheat bran, 10 g of sugar, and 10 g of calcium carbonate were evenly incorporated into all substrates, which were prepared on a dry weight basis. The pruned branches of Goji and Seabuck were collected from Nomuhong Town in Golmud City, Qinghai Province, while *Korshinsk peashrub* was gathered in Ledu District, Haidong City, Qinghai Province. Oak sawdust was collected from a local oak forest located in Ankang City, Shaanxi Province. A sawdust crusher was used to reduce the size of *the Lycium, Hippophae*, and *Korshinsk* branches to sawdust measuring 3–5 cm in size.

**Table 1:** Proportions of sawdust in the test cultivation matrix formulations.

Treatments	Contents of different sawdust/%				Wheat bran/%	Sugar/%	Light calcium carbonate/%
	LBP	HRP	KP	OS			
10%LBP	10	–	–	68	20	1	1
20%LBP	20	–	–	58	20	1	1
30%LBP	30	–	–	48	20	1	1
10%HRP	–	10	–	68	20	1	1
10%LBP-10%HRP	10	10		58	20	1	1
10%KP-10%LBP-10%HRP	10	10	10	48	20	1	1
10%KP		–	10	68	20	1	1
20%KP		–	20	58	20	1	1
30%KP		–	30	48	20	1	1
OS	–	–	–	78	20	1	1

KP, Korshinsk peashrub; LBP, *Lycium barbarum* pruning; HRP, *Hippophae rhamnoides* pruning. OS, Oak sawdust.

### 2.2 Spawn and substrate preparation

The strain “Qihe#1” was deposited and is maintained at the Edible Mushroom Research Center of the Qinghai Academy of Agricultural and Forestry Sciences. The isolates are preserved in a potato-dextrose-agar (PDA) medium at 4°C.

A wheat grain culture medium (85% wheat, 10% sawdust, 3% bran, 1% gypsum, 1% sucrose) was prepared for culturing the mycelium of *L. edodes* (21, 30). The wheat grains were steamed until the interior of the grains had no white core, after which they were filtered and mixed with the other ingredients. Each mixture (250 g fresh weight) was placed in a polypropylene bag and autoclaved at 121°C for 4 using a horizontal autoclave pot (Runjin, Shandong, China) RJA-1200 (hereafter referred to as “seed bag”). After cooling, the mycelium, pre-prepared in PDA medium, was inoculated into each seed bag. The inoculated bags were then placed under room temperature conditions to cultivate the mycelium for 3 weeks.

The conventional production formula for *L. edodes* (78% oak sawdust, 20% wheat bran, 1% sugar, and 1% light calcium carbonate) was used as the control. Different proportions of seabuckthorn, goji berry, and *C. intermedia* sawdust were added to replace the sawdust in the control formula, serving as the experimental treatment groups. The specific treatment formulas are detailed in Table 1. The materials were prepared in accordance with the specified formula proportions, ensuring uniformity through mixing. Subsequently, water was added to achieve a moisture content of 55–60%. Finally, lime or calcium carbonate was added to adjust the pH to a range of 7.0–7.5. Each mixture (1,000 g fresh weight) was placed in a polypropylene bag measuring 18 cm × 36 cm × 50 cm and sterilized at normal pressure for 18–20 h at 100°C using the YXQWF-C horizontal rectangular pressure steam sterilizer (Yixiang, Hubei, China; hereafter referred to as “stick’”). After sterilization, a 2% (W/W) spawn, previously prepared in a seed bag, was inoculated into the cooled stick and incubated at room temperature in the dark for 1 month to allow for colonization and hyphal growth.

### 2.3 Incubation and fruit induction

The stick of *L. edodes* culture should be maintained in complete darkness at a temperature of 20–22°C and a relative humidity of 50–60%. Throughout the entire growth and development cycle of *L. edodes*, mycelium development is categorized into four consecutive stages based on the research by Song et al. (31), as shown in Table 2. The conclusion of each stage was recorded as the number of days after spawning. The polyethylene bags were removed to induce fruiting bodies at the end of stage 3. Twenty-four hours later, the sticks were placed on shelves inside the fruiting room, which was set to a temperature of 16°C and a relative humidity of 90%, with scattered light provided. The number of days to harvest (HT) was also documented. Mushrooms were harvested in total two times (1st: F1 and 2nd: F2). At the conclusion of F1, the sticks were incubated again in a dark environment with a fruiting room temperature of 20–22°C and a relative humidity of 60%. During each mushroom harvest, the quantity and weight of fresh mushrooms in each stick (NM) were recorded. Additionally, the yield was assessed as the total weight of mushrooms harvested per stick (WM) during the two flushes. In the first flush, a sliding caliper was used to measure the mushroom pileus diameter.

**Table 2:** The four consecutive stages of shiitake mycelium development (31).

Stage	Term	Description
1	Mycelial growth stage	In the initial stage of growth, a thin layer of white hyphae in large numbers completely covers the stick until the culture substrate is fully utilized
2	Light-induced browning stage	The hardening mycelia sheet covers the whole substrate surface. Villous mycelium will gradually appear on the surface of the mycelium stick and converge to form a mycelium coat (called artificial bark). At the same time, brown pigment will be secreted to make the mycelium dark brown, which is a sign of mycelium maturity
3	Primordial formation stage	The mycelium knot gradually forms the primordium, and clumps of mycelia develop into a popcorn shape. At the same time, the mycelia stick develops a dark brown and dry outer protective layer
4	Fruiting body development stage	The stage of fruiting body formation lasts for 6 months

### 2.4 Nutritive composition of mushroom

Fresh and representative mushroom samples from each treatment of the first flush were selected to evaluate their nutritional composition. Each analysis was conducted three times. The ash content was determined by taking 5.0 g samples of mushrooms, placing them in a crucible, and heating them at 550°C in a muffle furnace for 24 h. The ash content was calculated as shown in Equation 1:


(1) $$\mathrm{Ash}\left(g\right)=M2\left(g\right)-M1\left(g\right)$$

M2: weight of the crucible containing ash.

M1: weight of an empty crucible.

The fat content (%) was assessed using the Soxhlet apparatus technique (32) by weighing 5.0 g of mushroom samples and placing them in the pre-weighed Soxhlet extractor flask (M1). A volume of 500 mL of petroleum ether was added to the flask, and the system was then heated for 10 h. Subsequently, the residue in the bottom flask was dried in a bain-marie at 100°C ± 5°C for 1 h, after which the fat remaining in the bottom flask was weighed (M2). The fat content was calculated as shown in Equation 2:


(2) $$\mathrm{Fat}\left(g\right)=M2\left(g\right)-M1\left(g\right)$$

M2: weight of the flask containing fat.

M1: weight of the empty flask of the extractor.

Crude fiber content (%) was determined according to NFSS (33) as the loss on ignition of the dried residue remaining after digesting a 5.0 g dry mushroom sample in 1.25% (w/v) H_2_SO_4_ and 1.25% (w/v) KOH. Crude protein content (N*6.25; %) was determined using the Kjeldahl method based on NFSS (34).

Amino acid content (%) was determined according to NFSS (35). First, 2.0 g of the sample was prepared and placed in a hydrolysis tube, followed by the addition of 10 mL hydrochloric acid (6 mol/L). The system was then frozen, vacuum-sealed, and filled before being hydrolyzed at 110°C for 22 h. After hydrolysis, the tubes were rinsed with water, and the rinsing waste liquid (V1) and hydrolysate (V2) were combined and diluted to a final volume of 50 mL. Subsequently, 1.0 mL of the hydrolysate was accurately measured. Following thorough sample preparation, which included concentration, washing, and drying, the processed hydrolysate was supplemented with 2.0 mL of sodium citrate solution. Amino acid content was analyzed using an amino acid analyzer, and concentrations of amino acids in the sample were calculated using the external standard method based on peak area. Amino acid content was calculated using the following Equation 3:


(3) $$\mathrm{Amino acid content}\left(g/100g\right)=\frac{C_{i}\times F\times V\times M}{2\times 10^{9}}\times 100$$

C_i_: The concentration in the sample solution (nmol/mL).

F: The dilution factor.

V: The volume of the hydrolysate transferred and diluted (mL).

M: The molar mass of the amino acid (g/mol).

The extraction and analysis of organic acids were conducted as follows: a 1.0 g sample was weighed in a centrifuge tube, and 10 mL of water was added and vortexed for 30 s. After ultrasonic extraction at room temperature for 30 min, the sample was centrifuged at 5,000 r/min for 5 min. The supernatant was mixed with 10 mL of ethanol and vortexed for another 30 s, then centrifuged again at 5,000 r/min for 5 min. The resulting supernatant was transferred to a chicken heart flask before being evaporated to dryness using rotary evaporation, redissolved in 5 mL of water, centrifuged at 10000 r/min for 5 min, filtered through a 0.22 μm membrane, and prepared for testing. The HPLC system utilized an LC-2030C Plus Athena-C18 column (4.6 mm × 250 mm, 5 μm) sourced from Shimadzu, based in Shanghai, China. To identify and quantify the organic acids, their retention times were compared to those of authentic standards supplied by Yuanye Bio-Technology Co., Ltd., also in Shanghai, China. Furthermore, the quantification of each sample was achieved by comparing its peak area against the calibration curve of the corresponding standard compound.

The extraction process for polyols and soluble sugars from the samples was conducted according to previously established methods. The LC-20AT HPLC system utilized an Athena NH2-RP (II) column (4.6 mm × 250 mm, 5 μm) paired with a 20A RID. The primary soluble sugars in the samples were fully resolved within a 40-min isocratic elution. The column was maintained at 40°C, with a mobile phase of acetonitrile: water (70: 30) at a flow rate of 1.0 mL/min, and an injection volume of 10 μL was employed. Each analyte was assessed against a reference sample from Yuanye (Shanghai) and quantified using the calibration curve of the respective authentic compounds.

### 2.5 Statistical analysis

Data analysis was conducted using a one-way analysis of variance (ANOVA), and means were compared using Duncan’s multiple range test at a *p*-value of <0.05 in SPSS 25.0. To explore the relationships between indicators during the growth stage of *L. edodes*, Pearson correlation analysis was conducted using the Correlation Plot function in Origin 2021. To mitigate the adverse effects of different measurement scales, the original data for each index were standardized using SPSS 25.0. Principal component analysis (PCA) was then applied to reduce dimensionality and simplify the data. Additionally, membership function analysis was used to standardize the indexes obtained from the principal component analysis. The membership function values and the comprehensive evaluation value (36) were calculated using the following Equations 4–6:


(4) $$\mu \left(X_{j}\right)=\frac{\left(X_{j}-X_{\text{min}}\right)}{\left(X_{\text{max}}-X_{\text{min}}\right)}$$

(5) $$W_{j}=\frac{P_{j}}{\sum_{j=1}^{n}P_{j}}$$

(6) $$D=\overset{n}{\underset{j=1}{\sum }}\left[U\left(X_{j}\right)\times W_{j}\right]$$

X_j_: The measured value of the j^th^ index.

X_max_: The maximum value of the j^th^ index.

X_min_: The minimum value of the j^th^ index.

W_j_: The importance degree of the j^th^ comprehensive indicator in all comprehensive indicators.

P_j_: The contribution rate of the j^th^ comprehensive index.

D: The comprehensive evaluation of different tested substrates.

## 3 Results

### 3.1 Mycelia growth and fruit body development of mushrooms

After the incubation period, the substrates showed dark-colored patches that eventually spread to cover the entire surface. According to the results of the one-way ANOVA (Table 3), there was no significant difference in the average number of days required to complete stages 1, 2, and 3 of mycelial growth on the tested substrates compared to the control, except for a significant reduction in stages 2 and 3 on 10%KP (reduced by 11.0 and 10.7 days, respectively). Conversely, the average number of days to reach stage 4 was significantly delayed in 20%KP and 30%KP compared to the OS (delayed by 34.67 and 35.17 days, respectively). Notably, the average number of days to reach stage 4 was reduced in the mixture 10%LBP-10%HRP compared to the OS (by 2 days). The duration was also significantly extended in the 20%KP and 30%KP treatments compared to OS treatment, with increases of 35.26 days and 35.88 days, respectively.

**Table 3:** The number of days to the mycelium development, fruit formation, and harvest in different experiments.

Treatments	Stage 1	Stage 2	Stage 3	Stage 4	Days to 1st harvest
10%LBP	28.17 ± 6.1ab	67.17 ± 2.6a	90.83 ± 3.3ab	107.5 ± 8.3d	109.48 ± 3.9d
20%LBP	26.83 ± 2.7ab	68.5 ± 7.1a	98 ± 2ab	111.5 ± 11 cd	114.15 ± 8.1 cd
30%LBP	25.67 ± 4.8ab	66.83 ± 9.7a	89.33 ± 3.6ab	113.83 ± 4 cd	117.48 ± 10.4 cd
10%HRP	31.67 ± 6.2a	63.83 ± 2.6ab	90.67 ± 3.9ab	123.5 ± 7.1c	119.95 ± 4.1c
10%LBP-10%HRP	27.33 ± 4.1ab	67 ± 4a	94.17 ± 3.7ab	103.67 ± 3.4b	129.97 ± 6.8b
10%KP-10%LBP-10%HRP	25.83 ± 3.7ab	62.17 ± 4.4ab	88 ± 6.7ba	114.33 ± 6.3c	120.39 ± 6.3c
10%KP	31.5 ± 4.1b	57 ± 2.1b	80.33 ± 10c	112.83 ± 10.2 cd	119.02 ± 9.2c
20%KP	26.83 ± 2.7ab	64.67 ± 4.1ab	92.67 ± 3ab	140.17 ± 5.8a	146.35 ± 5.7a
30%KP	25.17 ± 4.17b	69.33 ± 4.3a	90.33 ± 5.3a	140.67 ± 6a	146.97 ± 5.8a
OS	25.83 ± 5.9ab	68 ± 5.4a	91 ± 5.1ab	105.5 ± 9.3 cd	111.09 ± 9 cd

KP, Korshinsk peashrub; LBP, *Lycium barbarum* pruning; HRP, *Hippophae rhamnoides* pruning. OS, Oak sawdust. Means followed by lower-case letters indicate statistical difference according to Tukey (*p* < 0.05).

### 3.2 Production on tested substrates

Results in Table 4 showed that the average mushroom number was significantly reduced compared to the control, except for 10% HRP, 20% KP, and the mixture of 10% KP-10% LBP-10% HRP (by 8.3, 7.9, and 6.9, respectively). The mixture treatment of 10% KP-10% LBP-10% HRP significantly improved the average mushroom number compared to LBP alone (by 9.2) and KP alone (by 7.5). The average mushroom number for LBP ranged from 16.8 to 24.1, showing a statistical difference from the KP substrates (10, 20, and 30%, respectively). On the other hand, the weight of mushrooms was significantly lower than the control in all substrates. The average mushroom weight in the KP groups ranged from 235.37 g/kg to 252.27 g/kg, demonstrating a statistically significant increase compared to the LBP treatments, where the average weight ranged from 143.56 g/kg to 165.96 g/kg. Mixing LBP and HRP improved the weight of mushrooms compared to LBP alone (by 35.38 g/kg) and HRP alone (by 48.5 g/kg). Simultaneously, the weight of mushrooms increased correspondingly (by 9.11 g/kg and 7.79 g/kg, respectively) with the addition of KP. However, mixing LBP, KP, and HRP reduced the weight of mushrooms compared to KP alone (by 14.26 g/kg).

**Table 4:** The number of days to the mycelium development, fruit formation, and harvest in different culture medium experiments.

Treatments	NM	BY(g/kg)	SM(g)	BE (%)	Production of flush (%)		Production by each size group (%)		
					F1	F2	G1	G2	G3
10%LBP	24.1 ± 3.7bc	165.96 ± 0.16 g	6.86 ± 0.04f	33.3 ± 4.2 g	100	0	26.7 ± 0.3abc	73.3 ± 0.5a	0
20%LBP	16.8 ± 1d	143.56 ± 0.57i	7.35 ± 0.57e	28.7 ± 2.2i	100	0	20.0 ± 0.3bc	73.3 ± 0.4ab	6.7 ± 0.1b
30%LBP	20 ± 4.6 cd	150.81 ± 0.58 h	7.6 ± 0.05d	30.3 ± 8.2 h	100	0	0	100	0
10%HRP	33.7 ± 2.9a	152.84 ± 0.36 h	4.35 ± 0.09 g	30.6 ± 4.6 h	100	0	46.7 ± 1.0abc	53.3 ± 0.3abc	0
10%LBP-10%HRP	26.5 ± 6.2b	201.34 ± 1.05f	7.54 ± 0.05de	40.2 ± 2.0f	99.57	0.43	86.6 ± 0.2a	13.4 ± 0.2bc	0
10%KP-10%LBP-10%HRP	33.3 ± 1.3a	221.11 ± 0.24e	6.84 ± 0.05f	44.2 ± 3.1e	92.56	7.04	73.3 ± 1.5ab	26.7 ± 0.3bc	0
10%KP	25.8 ± 4.7b	235.37 ± 9.05d	9.07 ± 0.04c	46.1 ± 1.4d	100	0	73.3 ± 1.8ab	26.7 ± 0.6bc	0
20%KP	32.3 ± 1.3a	244.48 ± 0.25c	7.52 ± 0.02de	48.9 ± 3.6c	94.94	5.06	73.3 ± 0.4ab	26.7 ± 0.4bc	0
30%KP	23.6 ± 4.2bc	252.27 ± 0.77b	10.72 ± 0.01b	50.5 ± 3.8b	89.38	10.62	0	6.7 ± 0.1c	93.3 ± 1.5a
OS	25.4 ± 3.4b	311.28 ± 0.24a	12.29 ± 0.07a	62.2 ± 2.1a	100	0	0	6.7 ± 0.8c	93.3 ± 1.1a

NM, the number of mushrooms harvested; BY, weight of mushrooms per block (calculated by the fresh weight of mushrooms per block divided); SM, average fresh weight of a single mushroom from one block; BE, Biological efficiency [(fresh mushroom weight/substrate dry weight) × 100] (51); Production of flush: distribution of total weight mushrooms obtained in each harvest, estimated in percentage; Pileus size groups according to diameter: G1 < 5 cm, G2 5–9.9 cm, G3 10–15 cm; Values are mean, and ± are standard deviations. Means followed by lowercase letters indicate statistical difference, according to Tukey’s test (*p* < 0.05).

Furthermore, both the average fresh weight of mushrooms and the total biological efficiency were significantly lower than that of OS across all substrates (Table 4). The average biological efficiency (BE) for KP ranged from 46.1 to 50.5%, which was statistically higher than that of LBP (ranging from 28.7 to 33.3%). Mixing LBP and HRP improved biological efficiency compared to LBP alone (an increase of 6.9%) and HRP alone (an increase of 9.6%). Notably, the comparison of productive indicators between flush 1 and flush 2 showed that 20% KP, 30% KP, and 10% KP-10% LBP-10% HRP resulted in significantly higher production of the second flush compared to other substrates (with second flush ratios of 5.06, 10.62, and 7.04%, respectively).

Moreover, mushrooms produced by the substrate inoculated with KP, HRP, and LBP were mainly from the G1 and G2 groups, except that 30% LBP produced 100% mushrooms from the G2 group and 30% KP produced 6.7% mushrooms from the G2 group. The supplemented 30% KP produced larger mushrooms (the pileus diameter 10–15 cm) than the other tested substrates.

### 3.3 Nutritional composition of mushrooms

The analysis of the nutritional composition of mushrooms (Table 5) showed that the protein content of the fruit body was significantly higher in 10% LBP and 10% HRP (24.53 and 26.45%, respectively) compared to OS. Additionally, it was significantly improved in treatments containing LBP (10% LBP, 20% LBP, 30% LBP, and 10% LBP-10% HRP) compared to those containing KP (10% KP, 20% KP, and 30% KP), with the highest value recorded in 10% LBP (24.53%). The fiber content of the mushrooms was enhanced in all tested substrates compared to OS, with the highest values found in 10% HRP and 30% KP (10.38 and 10.46%, respectively). Ash content was significantly lower in most substrates compared to OS. Fat content was lower in mushrooms from most substrates compared to OS, except in 20% LBP, 30% LBP, 10% LBP-10% HRP, and 10% KP-10% LBP-10% HRP (0.90, 0.79, 0.79, and 0.91%, respectively), where it was significantly higher.

**Table 5:** Nutritional components analysis of the used sawdust and harvest fresh *L. edodes* in 10 different substrates.

Treatments	Crude protein (%)	Crude fiber (%)	Ash content (%)	Crude fat (%)
10%LBP	24.53 ± 0.31ab	10.03 ± 0.85ab	6.34 ± 0.04b	0.75 ± 0.02b
20%LBP	19.98 ± 1.21 cd	10.25 ± 0.20ab	4.03 ± 0.08f	0.90 ± 0.05a
30%LBP	21.49 ± 4.28bcd	9.97 ± 0.15ab	4.83 ± 0.07d	0.79 ± 0.02b
10%HRP	26.45 ± 0.63a	10.38 ± 0.32a	5.66 ± 0.04c	0.45 ± 0.03e
10%LBP-10%HRP	21.24 ± 0.68bcd	9.66 ± 0.52ab	5.48 ± 0.02c	0.79 ± 0.04b
10%KP-10%LBP-10%HRP	19.40 ± 0.84 cd	9.83 ± 0.57ab	4.61 ± 0.08de	0.91 ± 0.04a
10%KP	17.77 ± 1.07d	10.21 ± 0.83ab	4.36 ± 0.03e	0.64 ± 0.04c
20%KP	19.34 ± 2.83 cd	9.97 ± 0.78ab	4.58 ± 0.08de	0.79 ± 0.03b
30%KP	18.02 ± 3.88d	10.46 ± 0.19a	4.37 ± 0.02e	0.55 ± 0.01d
OS	23.43 ± 1.94abc	9.29 ± 0.29b	7.24 ± 0.55a	0.65 ± 0.04c

KP, Korshinsk peashrub; LBP, *Lycium barbarum* pruning; HRP, *Hippophae rhamnoides* pruning. OS, Oak sawdust. Values are mean, and ± are standard deviations. Means followed by lowercase letters indicate statistical difference, according to Tukey’s test (*p* < 0.05).

### 3.4 Amino acid composition of mushrooms

Table 6 shows the amino acid composition and content of shiitake mushroom fruiting bodies cultivated on various substrate formulations. Seventeen amino acids—aspartate, threonine, serine, valine, methionine, isoleucine, leucine, phenylalanine, lysine, glutamate, glycine, alanine, L-cysteine, tyrosine, histidine, arginine, and proline—were identified in shiitake mushrooms, while tryptophan was found at levels below the detection limit. The content of 15 amino acids across 8 substrates showed a significant decrease compared to the control. Specifically, the amino acid content in the 20% KP treatment group increased from 0.529 to 14.527, whereas it ranged from 0.032 to 3.854 in the 10% KP treatment. Conversely, the glutamate content of all test substrates was significantly lower than that of the OS, while the methionine levels in the 10% LBP, 20% LBP, and 30% LBP treatments were significantly higher compared to the control (1.411, 0.98, and 0.857, respectively).

**Table 6:** Amino acid composition analysis of *L. edodes* in 10 different substrates.

Type of amino acid	10%LBP	20%LBP	30%LBP	10%HRP	10%LBP-10%HRP	10%KP-10%LBP-10%HRP	10%KP	20%KP	30%KP	OS
Aspartate	1.975 ± 0.072de	1.859 ± 0.158ef	2.107 ± 0.01c	1.265 ± 0.114 g	1.809 ± 0.027f	1.961 ± 0.068de	2.461 ± 0.009b	4.525 ± 0.026a	1.332 ± 0.005 g	2.007 ± 0.046 cd
Threonine	1.515 ± 0.025c	1.375 ± 0.16e	1.511 ± 0.017c	0.961 ± 0.046 g	1.413 ± 0.025de	1.498 ± 0.034 cd	1.939 ± 0.011	3.644 ± 0.017ba	1.076 ± 0.005f	1.533 ± 0.006c
Serine	1.044 ± 0.014 cd	0.949 ± 0.056e	1.049 ± 0.025c	0.685 ± 0.035f	0.93 ± 0.002e	1.002 ± 0.017d	1.337 ± 0.004b	2.578 ± 0.017a	0.707 ± 0.004f	1.034 ± 0.013 cd
Valine	1.159 ± 0.003c	0.984 ± 0.032f	1.111 ± 0.005d	0.756 ± 0.009 g	1.011 ± 0.003e	1.096 ± 0.005d	1.425 ± 0.006b	3.253 ± 0.015a	0.155 ± 0.003 h	1.092 ± 0.005d
Methionine	1.705 ± 0.155a	1.274 ± 0.085b	1.151 ± 0.018c	0.192 ± 0.014ef	0.156 ± 0.016f	0.242 ± 0.046ed	0.372 ± 0.026d	0.167 ± 0.021f	0.221 ± 0.034ef	0.294 ± 0.072de
Isoleucine	0.721 ± 0.02c	0.602 ± 0.014f	0.696 ± 0.012 cd	0.443 ± 0.005 g	0.632 ± 0.004ef	0.657 ± 0.003de	0.822 ± 0.015b	1.691 ± 0.07a	0.438 ± 0.001 g	0.666 ± 0.003de
Leucine	1.851 ± 0.016c	1.524 ± 0.228e	1.823 ± 0.006 cd	1.233 ± 0.002f	1.683 ± 0.003d	1.812 ± 0.004 cd	2.344 ± 0.009b	4.495 ± 0.087a	1.194 ± 0.004f	1.82 ± 0.011 cd
Phenylalanine	1.038 ± 0.002c	0.909 ± 0.073e	0.971 ± 0.006d	0.679 ± 0.003f	0.986 ± 0.002d	0.973 ± 0.003d	1.297 ± 0.006b	2.362 ± 0.011a	0.667 ± 0.004f	1.031 ± 0.001c
Lysine	0.819 ± 0.002f	0.774 ± 0.002 h	0.808 ± 0.003 g	0.543 ± 0.002i	0.825 ± 0.001e	0.835 ± 0d	1.107 ± 0.004b	1.808 ± 0.006a	0.537 ± 0.002j	0.865 ± 0.006c
Glutamate	7.595 ± 0.063ab	7.95 ± 0.336ab	7.965 ± 0.115ab	4.407 ± 0.107c	8.246 ± 0.013a	7.079 ± 0.084ab	7.396 ± 4.178ab	4.503 ± 0.114c	5.65 ± 0.01bc	8.721 ± 0.089a
Glycine	0.697 ± 0.004c	0.621 ± 0.014e	0.68 ± 0.011 cd	0.427 ± 0.001 g	0.66 ± 0.003d	0.607 ± 0.024e	0.798 ± 0.029b	1.556 ± 0.006a	0.451 ± 0.002f	0.662 ± 0.004d
Alanine	1.115 ± 0.014c	0.952 ± 0.023f	1.052 ± 0.036d	0.674 ± 0.005 g	1.034 ± 0.015d	0.983 ± 0.017ef	1.308 ± 0.039b	2.296 ± 0.002a	0.673 ± 0.007 g	1.016 ± 0.01de
L-Cysteine	0.177 ± 0.017c	0.135 ± 0.007de	0.125 ± 0.002e	0.153 ± 0.002d	0.125 ± 0.001e	0.186 ± 0.007c	0.247 ± 0.019b	0.114 ± 0.003e	0.772 ± 0.029a	0.198 ± 0.009c
Tyrosine	0.512 ± 0.002c	0.422 ± 0.025 g	0.467 ± 0.003 cd	0.302 ± 0.004 h	0.448 ± 0.004f	0.454 ± 0.003ef	0.587 ± 0.004b	1.119 ± 0.005a	0.306 ± 0.001 h	0.488 ± 0.001 cd
Histidine	1.769 ± 0.004b	1.775 ± 0.006b	1.712 ± 0.006d	1.426 ± 0.008 g	1.747 ± 0.015c	1.623 ± 0.008e	1.783 ± 0.008b	2.28 ± 0.016a	1.567 ± 0.013f	1.751 ± 0.009c
Arginine	0.701 ± 0.012c	0.696 ± 0.163c	0.676 ± 0.064c	0.424 ± 0.01d	0.704 ± 0.009c	0.752 ± 0.144bc	0.886 ± 0.01b	1.484 ± 0.016a	0.525 ± 0.082d	0.783 ± 0.005bc
Proline	5.047 ± 0.21de	5.667 ± 0.123e	5.112 ± 0.292de	4.005 ± 0.63e	8.196 ± 0.866c	8.527 ± 1.67c	12.268 ± 0.275b	22.941 ± 0.064a	5.656 ± 0.809d	8.414 ± 0.447c
EAA	11.8 ± 0.265c	10.223 ± 0.366e	11.167 ± 0.058d	6.753 ± 0.212 g	9.44 ± 0.02f	10.03 ± 0.121e	13.033 ± 0.058b	24.433 ± 0.115a	6.323 ± 0.021 h	10.267 ± 0.058e
NEAA	17.614 ± 0.274de	18.219 ± 0.268de	17.787 ± 0.417de	11.818 ± 0.69f	21.16 ± 0.855c	20.211 ± 1.808 cd	25.274 ± 4.075b	36.293 ± 0.046a	15.601 ± 0.769e	22.033 ± 0.444c
TAA	29.44 ± 0.504d	28.468 ± 0.664d	29.014 ± 0.397d	18.576 ± 0.866f	30.605 ± 0.866 cd	30.287 ± 1.958 cd	38.379 ± 4.082b	60.816 ± 0.08a	21.927 ± 0.75e	32.375 ± 0.385c
SAA	11.188 ± 0.216d	11.338 ± 0.266d	11.115 ± 0.225d	8.178 ± 0.692e	13.98 ± 0.83c	14.239 ± 1.732c	19.434 ± 0.244b	35.295 ± 0.092a	10.13 ± 0.805d	14.411 ± 0.447c
BAA	6.136 ± 0.165b	5.081 ± 0.37e	5.458 ± 0.055d	3.048 ± 0.019 h	4.187 ± 0.025 g	4.56 ± 0.18f	5.849 ± 0.018c	11.09 ± 0.037a	2.533 ± 0.106i	4.655 ± 0.057f
MSAA	10.389 ± 0.136a	10.583 ± 0.493a	10.88 ± 0.115a	6.215 ± 0.219b	10.88 ± 0.035a	9.875 ± 0.152a	10.965 ± 4.171a	10.835 ± 0.088a	7.518 ± 0.015b	11.593 ± 0.14a
ARAA	1.55 ± 0.003c	1.331 ± 0.098e	1.437 ± 0.009d	0.982 ± 0.006f	1.434 ± 0.005d	1.427 ± 0.006d	1.885 ± 0.004b	3.481 ± 0.016a	0.973 ± 0.003f	1.519 ± 0.011c
EAA/TAA	40.20% ± 0.10%a	36.00% ± 0.56%c	38.70% ± 0.61%ab	36.40% ± 0.89%bc	30.87% ± 0.85%ef	33.33% ± 1.69%de	34.43% ± 3.87%cd	40.33% ± 0.12%a	28.90% ± 1.04%f	31.93% ± 0.55%de
EAA/NEAA	67.17% ± 00.31%a	56.23% ± 1.37%cd	63.13% ± 1.62%ab	57.27% ± 2.22%bc	44.67% ± 1.75%fg	50.07% ± 3.88def	52.87% ± 9.31%cde	67.57% ± 0.4%a	40.63% ± 2.11%g	46.93% ± 1.25%ef

KP, Korshinsk peashrub; LBP, *Lycium barbarum* pruning; HRP, *Hippophae rhamnoides* pruning. OS, Oak sawdust. Values are mean, and ± are standard deviations. EAA, essential amino acid; NEAA, nonessential amino acid; TAA, total amino acids; SAA, sweet amino acid; BAA, bitter amino acid; MSAA, MSG-like amino acid; ARAA, aromatic amino acid. Means followed by lowercase letters indicate statistical difference, according to Tukey’s test (*p* < 0.05).

Furthermore, the content of sweet-tasting amino acids significantly increased in 10%KP and 20%KP compared to the OS (by 5.023 and 20.884, respectively). The content of bitter-tasting amino acids and aromatic amino acids also showed significant increases in 10%KP (by 1.194 and 0.366, respectively) and 20%KP (by 6.435 and 1.962, respectively) when compared to the OS. In contrast, the MSG-like amino acid content significantly decreased in all tested substrates compared to the OS.

Moreover, the total content of essential amino acids in the 10% KP and 20% KP treatments was significantly greater compared to the OS (by 2.711 and 14.166, respectively). Similarly, the total content of nonessential amino acids in the 10% KP and 20% KP treatments was also significantly greater compared to the OS (by 3.241 and 14.26, respectively). The EAA/TAA ratio of the LBP group improved significantly by 23.66 to 30.43% compared with the OS, and the EAA/NEAA ratio also rose by 38.66 to 54.86%.

### 3.5 The soluble sugars and organic acids contents in mushrooms

The contents of soluble sugars and organic acids in the fruiting bodies of *L. edodes* cultivated on different substrates are shown in Table 7. Arabitol content was higher in mushrooms cultivated on substrates with a higher proportion of 10% LBP, 20% KP, and the mixture of 10% KP-10% LBP-10% HRP, showing a significant difference compared to the other groups (*p* < 0.05). The 30% LBP group and the mixture of 10% KP-10% LBP-10% HRP exhibited higher glucose values compared to the OS (p < 0.05). The addition of 10% LBP to the substrate also resulted in a significantly increased trehalose content compared to OS (p < 0.05). In total, nine organic acids were detected in all samples. Statistical analysis showed that the content of mushrooms in the tested substrates was significantly different (Table 7). The tartaric acid content in mushrooms cultured in the 30% LBP treatment group was the highest (0.25 mg/g), whereas the OS group had the lowest (0.04 mg/g). The 20% LBP treatment provided the least amount of formic acid (0.13 mg/g), while the 20% KP treatment yielded the highest content (5.59 mg/g). The malic acid content in shiitake mushrooms ranged from 0.89 to 1.31 mg/g. Interestingly, the addition of LBP and KP to the substrate enhanced the synthesis of acetic acid in shiitake mushrooms, which was not detected in the OS. The citric acid content in the samples from the 10% LBP treatment group was the highest (12.66 mg/g), followed by the 10% KP-10% LBP-10% HRP treatment group (11.33 mg/g) and the 10% LBP-10% HRP treatment group (10.32 mg/g). For the fumaric acid content in *L. edodes* fruiting bodies, the 10% LBP-10% HRP treatment group showed the highest level (3.88 mg/g), followed by the 10% LBP group (3.88 mg/g) and the 30% LBP group (2.86 mg/g). Additionally, it was suggested that the addition of 30% LBP and 20%KP had a significant influence on the contents of succinic acid and butyric acid in *L. edodes*.

**Table 7:** The contents of soluble sugars and organic acids in *L. edodes* samples of different cultivation substrates.

	Arabitol (mg/g)	Glucose (mg/g)	Trehalose (mg/g)	Tartaric acid (mg/g)	Formic acid (mg/g)	Malic acid (mg/g)	Acetic acid (mg/g)	Citric acid (mg/g)	Fumaric acid (mg/g)	Succinic acid (mg/g)	Propionic acid (mg/g)	Butyric acid (mg/g)
10%LBP	35.18 ± 1.63a	1.82 ± 0.07c	11.94 ± 0.19a	0.08 ± 0.01c	2.18 ± 0.01d	1.29 ± 0.02b	0.71 ± 0.01a	12.66 ± 0.12a	3.88 ± 0.05b	0.87 ± 0.01a	0.2 ± 0.01 g	0.15 ± 0.01d
20%LBP	0	0.95 ± 0.02 h	0	0.04 ± 0.01 g	1.65 ± 0.01f	0.89 ± 0.01i	0.29 ± 0.01d	6.70 ± 0.01 g	0.97 ± 0.02 h	0.40 ± 0.01 g	0.34 ± 0.01e	0.06 ± 0.01i
30%LBP	11.54 ± 0.34e	3.39 ± 0.16a	8.95 ± 0.01d	0.25 ± 0.01a	0.13 ± 0.01j	1.06 ± 0.01 g	0.48 ± 0.01b	8.59 ± 0.01e	2.86 ± 0.04c	0.87 ± 0a	0.35 ± 0.01d	0.80 ± 0.01a
10%HRP	17.6 ± 0.07d	1.66 ± 0.01d	4.00 ± 0.05 h	0.09 ± 0.01b	0.35 ± 0.01i	0.98 ± 0.01 h	0	4.19 ± 0.01j	1.22 ± 0.02 g	0.87 ± 0.01a	0.06 ± 0.01j	0.27 ± 0.01c
10%LBP-10%HRP	12.51 ± 0.05e	1.50 ± 0.01f	11.32 ± 0.01c	0.05 ± 0.01f	1.94 ± 0.03e	1.27 ± 0.01c	0	10.32 ± 0.04c	4.57 ± 0.06a	0.57 ± 0.01c	0.40 ± 0.01c	0.05 ± 0.01j
10%KP-10%LBP-10%HRP	27.4 ± 0.14b	2.67 ± 0.04b	11.82 ± 0.07a	0.04 ± 0.01 g	1.35 ± 0.01 h	1.14 ± 0.01e	0	11.33 ± 0.03b	1.49 ± 0.02f	0.58 ± 0.01c	0.65 ± 0.01b	0.08 ± 0.01 g
10%KP	12.71 ± 0.11e	0.83 ± 0.01i	5.57 ± 0.05 g	0.07 ± 0.01d	1.51 ± 0.01 g	1.08 ± 0.01f	0.12 ± 0.01e	4.39 ± 0.02i	2.23 ± 0.1d	0.50 ± 0.01f	0.19 ± 0.01 h	0.08 ± 0.01f
20%KP	23.49 ± 5.74c	1.00 ± 0.02 h	8.62 ± 0.02e	0.05 ± 0.01f	5.59 ± 0.02a	1.19 ± 0.01d	0.37 ± 0.01c	7.02 ± 0.01f	0	0.53 ± 0.01e	0.16 ± 0.01i	0.07 ± 0.01 h
30%KP	12.7 ± 0.03e	1.31 ± 0.03 g	6.28 ± 0.06f	0.06 ± 0.01e	3.71 ± 0.01b	1.31 ± 0.01a	0.09 ± 0.01f	5.65 ± 0.02 h	1.88 ± 0.03e	0.65 ± 0.01b	0.31 ± 0.01f	0.31 ± 0.01b
OS	12.74 ± 0.11e	1.55 ± 0.03e	11.61 ± 0.15b	0.04 ± 0.01 g	3.48 ± 0.01c	1.06 ± 0.01 g	0	9.34 ± 0.05d	0.79 ± 0.01i	0.55 ± 0.01d	0.72 ± 0.01a	0.11 ± 0.01e

KP, Korshinsk peashrub; LBP, *Lycium barbarum* pruning; HRP, *Hippophae rhamnoides* pruning. OS, Oak sawdust. Values are mean, and ± are standard deviations. Means followed by lowercase letters indicate statistical differences, according to Tukey’s test (*p* < 0.05).

### 3.6 Correlation between productive indicators

**Figure 1:**
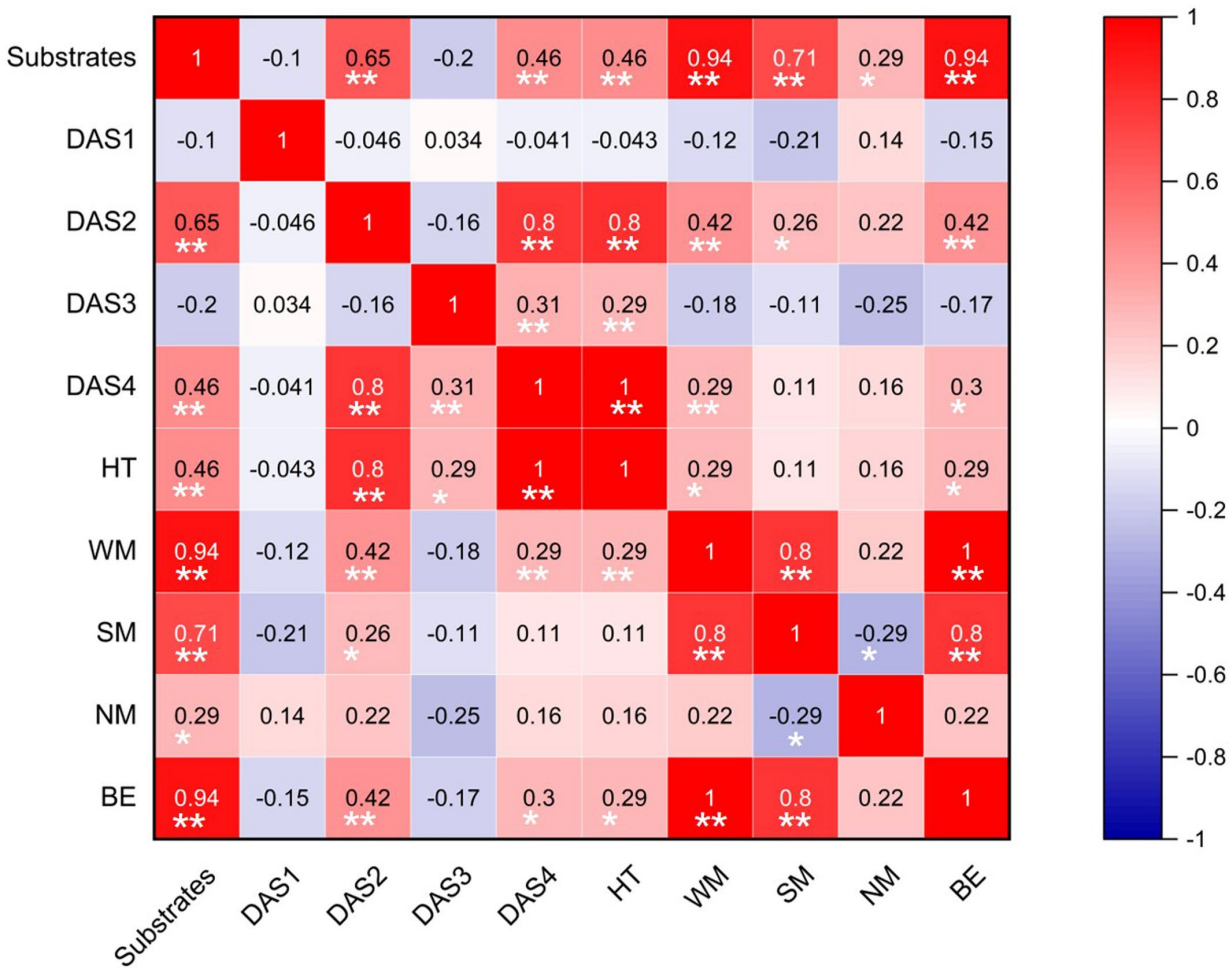
Significant correlations analysis of growth indicators of *Lentinus edodes*.

The results of the correlation analysis (Figure 1) showed that the substrates were positively correlated with seven growth indicators, whereas DAS1 and DAS3 exhibited a negative correlation. The weight of the mushroom (WM) was significantly influenced by DAS2, DAS4, HT, SM, and BE (*p* < 0.01). A negative correlation was found between NM and SM (*p* < 0.05). The harvest time was significantly impacted by DAS2 and DAS4 (*p* < 0.01). Conversely, there was a significant negative correlation between the cultivation (*p* < 0.05) substrate and glucose, tartaric acid, acetic acid, citric acid, fumaric acid, succinic acid, and butyric acid (Supplementary Figure 1). Among the three soluble sugars, a highly significant and strong positive correlation existed between arabiol and trehalose (*p* < 0.01). Regarding the nine organic acid components, there were extremely significant strong positive correlations between tartaric acid and butyric acid, succinic acid and tartaric acid, and succinic acid and butyric acid (with correlation coefficients of 0.95, 0.66, and 0.66, respectively; *p* < 0.01). Furthermore, glucose showed extremely significant positive correlations with tartaric acid and butyric acid, respectively.

**Figure 2:**
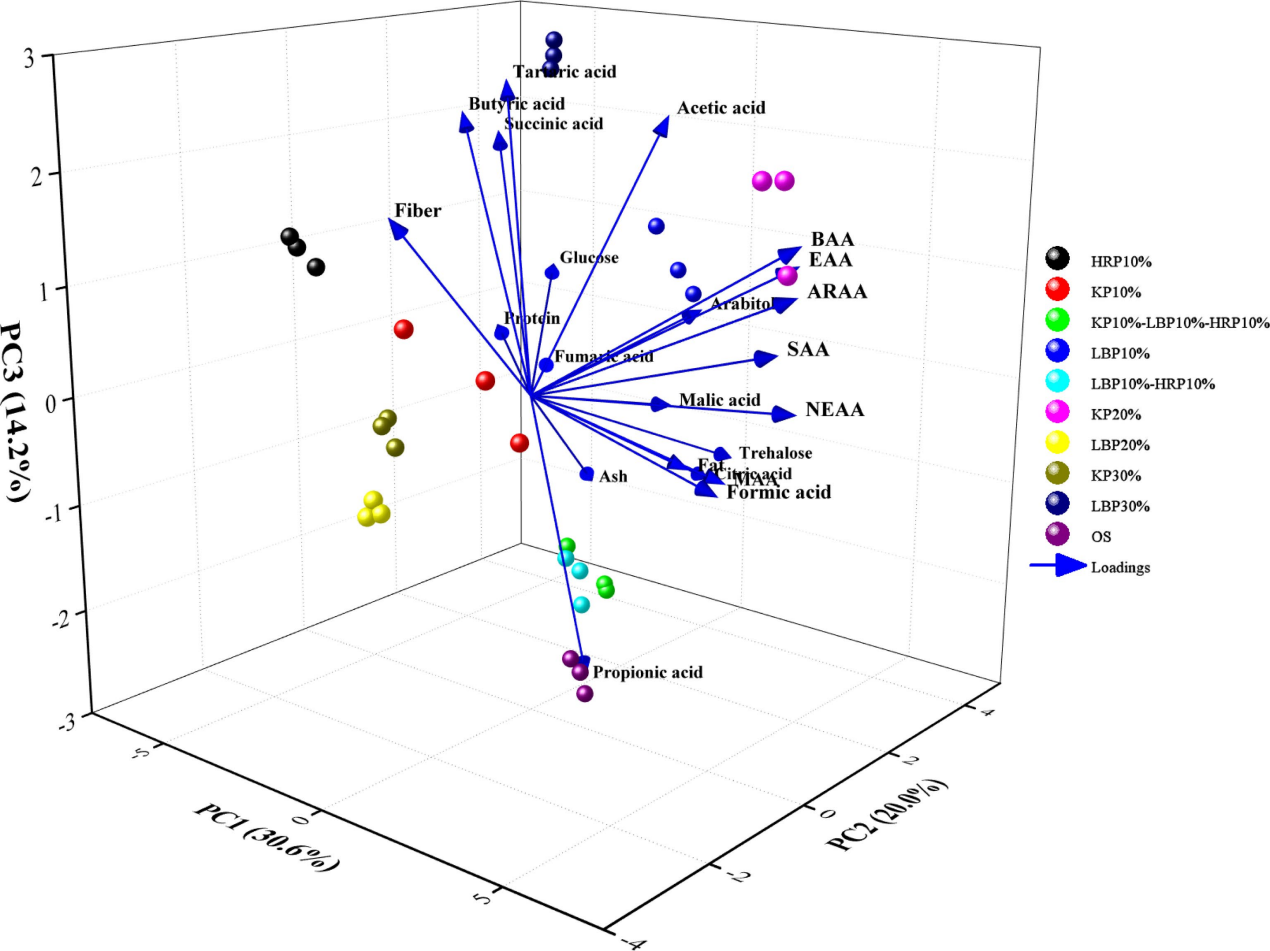
Principal components of quality indicators of *L. edodes*.

Considering that there are more than 16 indicators and varying degrees of correlation among them, principal component analysis (PCA) was used to simplify and reduce the dimensionality of each indicator. The original 16 indicators were transformed into six independent, comprehensive indicators based on the principle that the characteristic eigenvalue of a principal component is greater than 1.00 and the cumulative contribution rate exceeds 80% (Table 8). The cumulative contribution rate of these six principal components was 88.16%, indicating that the six components can retain 88.16% of the information from the original index, which adequately explains the quality of shiitake mushrooms. The quality indicators for the principal components of *L. edodes* are shown in Figure 2. The factors NEAA, SAA, and ARAA significantly influenced the first principal component (PC1) with loading coefficients of 0.369, 0.364, and 0.356, respectively, suggesting that PC1 primarily reflects amino acid-related information. The second principal component (PC2) was predominantly influenced by trehalose, citric acid, arabinol, and glucose, with loading coefficients of 0.415, 0.410, and 0.311, indicating that PC2 is mainly associated with soluble sugar content. The third principal component (PC3) was significantly affected by tartaric acid, acetic acid, and propionic acid. Among these, tartaric acid and acetic acid exhibited larger positive loading values, while propionic acid showed a larger negative loading value (loading values were 0. 376, 0. 343, and-0.430, respectively), meaning that PC3 primarily reflects organic acid content. Subsequently, the membership function values (μ_xj_) and comprehensive evaluation values (D) for the six principal component indicators were calculated, as detailed in Table 9. The results indicated that the comprehensive evaluation values for the groups 20% KP, 10% LBP, and 10% KP-10% LBP-10% HRP ranked among the top three, with D values of 0.67, 0.60, and 0.54, respectively. These findings suggest that shiitake mushrooms cultivated using these three substrates exhibit a higher nutritional value. Furthermore, among the 10 substrates evaluated, the groups 20% KP, 10% LBP, and 10% KP-10% LBP-10% HRP were identified as the optimal substrates for mushroom cultivation.

**Table 8:** Eigenvalue and loading coefficients of principal components.

	PC1	PC2	PC3	PC4	PC5	PC6
Ash	−0.070	0.240	−0.185	−0.268	0.441	−0.053
Fat	0.148	0.153	−0.103	0.421	−0.196	−0.313
Protein	−0.156	0.132	0.046	−0.226	0.458	−0.351
Fiber	−0.078	−0.191	0.248	−0.137	−0.279	−0.149
EAA	0.348	0.073	0.211	0.017	0.093	−0.015
NEAA	0.370	0.039	0.039	0.049	0.051	0.116
SAA	0.364	0.001	0.118	−0.034	0.052	0.147
BAA	0.334	0.101	0.229	0.037	0.099	−0.100
MAA	0.187	0.179	−0.119	0.316	0.093	−0.155
ARAA	0.357	0.055	0.177	−0.022	0.087	0.042
Arabitol	0.062	0.311	0.072	−0.337	−0.069	−0.061
Glucose	−0.183	0.300	0.101	0.262	0.057	0.229
Trehalose	0.051	0.416	−0.164	−0.113	−0.012	0.243
Tartaric acid	−0.163	0.163	0.376	0.263	0.076	0.213
Formic acid	0.294	−0.012	−0.068	−0.239	−0.019	0.223
Malic acid	0.062	0.239	−0.048	−0.313	−0.468	0.263
Acetic acid	0.055	0.232	0.344	0.052	−0.094	−0.378
Citric acid	0.014	0.411	−0.201	0.052	−0.108	−0.211
Fumaric acid	−0.153	0.246	−0.028	−0.020	−0.402	−0.189
Succinic acid	−0.233	0.235	0.292	−0.191	0.091	0.061
Propionic acid	−0.005	0.137	−0.431	0.252	0.111	0.224
Butyric acid	−0.201	0.114	0.335	0.224	0.061	0.355
Eigenvalue	6.729	4.408	3.12	2.388	1.552	1.197
Percentage of variance (%)	30.59	20.036	14.182	10.85	7.057	5.445
Cumulative (%)	30.59	50.626	64.809	75.664	82.72	88.166

KP, Korshinsk peashrub; LBP, *Lycium barbarum* pruning; HRP, *Hippophae rhamnoides* pruning. OS, Oak sawdust. Values are mean values. EAA, essential amino acid; NEAA, nonessential amino acid; TAA, total amino acids; SAA, sweet amino acid; BAA, bitter amino acid; MSAA, MSG-like amino acid; ARAA, aromatic amino acid.

**Table 9:** Membership function analysis of quality indicators of *L. edodes* with different cultivation substrates.

	X1	X2	X3	X4	X5	X6	U1	U2	U3	U4	U5	U6	D value	Ranking
10%LBP	−0.84	0.89	1.48	1.83	−0.42	0.8	0.17	0.78	1	1	0.42	0.8	0.6	2
20%LBP	0.06	−1.35	−0.55	1.33	−0.09	−1.71	0.4	0	0.34	0.84	0.52	0	0.33	9
30%LBP	0.07	0.69	−0.92	0.53	0.61	0.52	0.41	0.71	0.21	0.58	0.73	0.71	0.51	4
10%HRP	−1.48	−0.93	0.58	−1.24	−1.27	−0.13	0	0.15	0.71	0	0.16	0.51	0.19	10
10%LBP-10%HRP	0	0.65	−0.97	−0.09	0.9	−0.24	0.39	0.7	0.2	0.37	0.82	0.47	0.46	5
10%KP-10%LBP-10%HRP	−0.19	1.51	0.79	−1.09	0.6	−1.61	0.34	1	0.78	0.05	0.73	0.03	0.54	3
10%KP	0.46	−0.99	0.13	0.2	0.39	−0.06	0.51	0.13	0.56	0.47	0.66	0.53	0.43	6
20%KP	2.32	−0.26	1.13	−0.47	−0.41	0.45	1	0.38	0.89	0.25	0.42	0.69	0.67	1
30%KP	−0.64	−0.89	−0.08	−0.71	1.48	1.41	0.22	0.16	0.49	0.17	1	1	0.35	8
OS	0.24	0.7	−1.58	−0.29	−1.78	0.57	0.45	0.72	0	0.31	0	0.73	0.4	7

## 4 Discussion

### 4.1 Feasibility of using KP/HRP/LBP as a substrate for cultivating shiitake mushrooms

Cultivating edible mushrooms has proven to be an effective biological method for transforming agricultural and forestry byproducts. This study comprehensively evaluates the feasibility of using fruit tree pruning residues as substrates for cultivating *L. edodes*. Additionally, we analyzed and summarized the effects of different addition ratios of *Korshinsk* peashrub (KP), *Hippophae rhamnoides* pruning (HRP), and *Lycium barbarum* pruning (LBP) on the yield, amino acid, and protein contents of *Lentinus edodes*. Our research results demonstrate that utilizing sawdust derived from discarded branches for shiitake mushroom cultivation is a practical alternative. Both sawdust obtained solely from forest waste and partially mixed sawdust can be effectively employed in shiitake mushroom cultivation. However, it should be noted that the proportions of these additives can significantly influence mycelium growth and the development of fruiting bodies (Tables 3, 4). During the growth process of shiitake mycelium, extracellular enzymes such as cellulase, hemicellulase, and ligninase are secreted, playing a crucial role in degrading the lignocellulosic components present in the substrate (37). Lignin is tightly bound within the cell wall of lignocellulose materials, serving as a protective shield for cellulose and hemicellulose, making them less vulnerable to degradation by enzymes (38). Therefore, mushrooms must first decompose lignin before proceeding to degrade cellulose (including hemicellulose and cellulose) (39, 40). The higher the proportion of lignin in the initial substrate mixture, the lower the bioavailability of that substrate (41, 42).

In light of the previous remarks, the study demonstrated that a 10% proportion of KP led to the most rapid mycelium development compared to OS. Moreover, both quicker mycelial spread and fruiting body formation were achieved on the substrate containing 10% KP, 10% LBP, and 10% HRP, in contrast to those on substrates with only 10% LBP or 10% HRP. This phenomenon may be attributed to the presence of 10% KP in these substrates. Particularly, compared to the substrate containing 10% KP, the browning of mycelium and the formation of primordium of *Lentinus edodes* were delayed by 10 days and 10 days, respectively, on the substrate with 10% LBP. Moreover, relative to the substrate with 10% KP, these processes were delayed by 6 days for mycelium browning and 10 days for primordium formation on the substrate with 10% HRP. It is evident that the physical environment for mycelial growth is dependent on the specific characteristics of the substrate. Previous studies have highlighted the significant role that KP plays as a substrate component in mushroom cultivation (22, 24, 43). In the context of KP substrates, their structural characteristics mitigate the compaction effect that occurs during mycelial growth compared to substrates composed of sawdust. This reduction in compaction facilitates the hydration process of the KP substrates through regular irrigation (44). Furthermore, in contrast to oak sawdust commonly employed in commercial production, the fruiting bodies of shiitake mushrooms could be harvested 2.1 days earlier from the substrate composed of 10% LBP and 10% HRP. This earlier harvest shortened the production cycle of shiitake mushrooms and consequently brought substantial economic benefits.

Generally, growers can harvest 0.3 kg to 0.5 kg of fresh shiitake mushrooms (with a biological efficiency ranging from 30 to 50%) from 1 kg of dry substrate (45). Except for the 10% LBP and 10% HRP, the biomass yields of the other tested substrates were similar, ranging from 201.34 g/kg for the substrate with 10% LBP and 10% HRP to 311.28 g/kg for oak sawdust. The corresponding bioefficiency fell within the range of 40.2 to 62.2% (Table 4). At the experimental scale, Yu et al. (39) documented a higher bioefficiency of 80.8% using oak sawdust. However, Leifa et al. (46) reported diverse bioefficiencies that varied from 78.4 to 85.8% across different lignocellulosic substrates. An important factor affecting the growth and fruiting of edible fungi is the carbon-to-nitrogen (C/N) ratio of the growth substrate (47, 48). It should be noted that the minimum C/N ratio necessary for the growth of shiitake mushrooms is 25:1, while the maximum C/N ratio is 55:1 (49). Desisa et al. (9) found that the total yield of shiitake mushrooms was relatively higher (20.96 mg/g) on substrates characterized by low carbon-to-nitrogen ratios. However, in the current experiment, higher total yields were obtained on substrates with higher C/N ratios. These basic findings align with the results of Sassine’s (50) research on growing shiitake mushrooms by utilizing oak acorns, vineyard pruning, and living pruning.

### 4.2 The nutrition of shiitake mushrooms is influenced by KP/HRP/LBP substrate

The nutritional content of fruiting bodies is strongly influenced by both the composition of the cultivation substrate (51–54) and the specific cultivated strain (55). The protein content of Qihe #1 shiitake mushrooms cultivated in the tested cultivation media ranged from 17.77 to 26.45% (Table 5).

This range was higher than the 13.7–19.6% and 12.4–17.2% reported by Desisa et al. (9) and Gaitan-Hernandez et al. (9), respectively, for shiitake mushrooms grown on agricultural waste, but lower than the 20–23% reported by Rahman and Choudhury (56). This range was higher than the 13.7–19.6% and 12.4–17.2% reported by Desisa et al. (9) and Gaitan-Hernandez et al. (9) for shiitake mushrooms cultivated on agricultural waste and lower than the 20–23% reported by Rahman and Choudhury (56). The protein content in this study was similar to that of milk (25.2%) (57) and significantly higher than that of vegetables (58).

Elkanah et al. (59) reported that the high protein content of shiitake mushrooms cultivated in a culture medium supplemented with wheat bran may be due to the rich carbon and nitrogen levels in the medium. From a nutritional perspective, mushrooms are considered a valuable source of protein (53). This aligns with the hypothesis that mushrooms could serve as an effective substitute for meat, given that their nutritional value is comparable to that of numerous plant species (60, 61).

Currently, efforts are being made to explore protein sources that can satisfy the nutritional requirements of the world’s growing population (62). To date, a fitting approach has been suggested in which various wastes can be used to cultivate protein-rich mushrooms, thus enhancing the value of agro-forestry byproducts.

Mushroom fiber is mostly soluble and can be absorbed by the human intestine, while plant fiber mainly consists of cellulose or hemicellulose, which the human body cannot utilize (63). These fibers are beneficial to human health as they not only aid digestion but also act as prebiotics for gut microbes, helping to prevent disease (64). Furthermore, mushroom fibers such as chitin and *β*-glucan possess anti-obesity, anti-diabetes, and anti-hypertension properties. As a result, they are utilized in biomedicine for anti-inflammatory, anti-allergic, anti-cancer, and immunomodulatory applications (65, 66). The fiber content in mushrooms was enhanced in the tested substrates compared to OS, peaking at 10.26% in the substrate with 30% KP (Table 5). Moreover, this component was significantly higher in the substrate with 10% HRP compared to those with 30% LBP, 10% LBP-10% HRP, and 10% KP-10% LBP-10% HRP, which had fiber contents of 9.97, 9.66, and 9.83%, respectively. In contrast, shiitake mushrooms grown exclusively on rice straw exhibited the lowest crude fiber content at 1.5% (13). Producing fiber-rich mushrooms can meet the health and nutritional needs of various consumers, allowing them to be transformed into numerous innovative food products within the industry, thereby broadening the application range of mushroom products. The fat content of shiitake mushrooms cultivated in the 10 tested substrates was significantly lower, ranging from 0.45 to 0.91%, which is well below the 3–4% levels previously reported by Rahman and Choudhury (56). This suggests that shiitake mushrooms grown in these substrates are a low-calorie food source. The role of fat in the human body includes generating energy for muscles and bodily functions, as well as aiding in the digestion and absorption of nutrients (59). Therefore, the high nutritional value of shiitake mushrooms, which contain very little fat (less than 1.22%) and no cholesterol, has attracted global interest in this macro fungus (12, 67).

One of the most notable characteristics of shiitake mushrooms is their remarkable ability to enhance flavor, a quality attributed to the presence of volatile and non-volatile components in the fruiting bodies (68). The levels of sweet and aromatic amino acids in the 20%KP group increased significantly compared to the control group, with an increase ranging from 0.529 to 14.527 (Table 6). The presence of amino acids such as glutamic acid (Glu), aspartic acid (Asp), and monosodium glutamate (MSG) contributes to the perception of a salty flavor, while specific amino acid sequences such as Cys-Met, Glu-Pro-Glu, and Gly-Cys-Gly are crucial for the umami flavor of shiitake mushrooms (69–72). After LBP was added to the culture medium, the methionine content in the fruiting body increased remarkably. In particular, the content with the addition of 10% LBP was 5.79 times that of the control group. Methionine is a sulfur-containing amino acid that serves as a vital raw material in synthesizing the flavor of cooked meat through the Maillard reaction (73). These amino acids contribute to the overall taste experience and selectivity of shiitake mushrooms, making them a valuable ingredient in various culinary applications and food preparations.

Soluble sugar is one of the key substances that contribute to the flavor of shiitake mushrooms. It can interact with compounds such as amino acids and influence the development of sweet and caramel flavors during processing (68). The results showed that trehalose, arabinose, and glucose were the primary soluble sugars in the 10 tested mushrooms (Table 7). However, the total soluble sugar levels (19.11–48.94 mg/g) of the tested shiitake mushrooms in this study were significantly lower than the 78.65–126.1 mg/g reported by Chen et al. (74) and the 82.98–127.30 mg/g reported by Li et al. (75). The reasons for this difference may include the different strains of shiitake mushrooms and variations in cultivation substrates. During the growth of edible fungi, organic acids are closely associated with the metabolic processes involved in synthesizing phenols, amino acids, esters, and aromatic compounds. To some extent, the types and amounts of organic acids influence the formation of the unique flavor of the fruiting body (68). It was found that the primary organic acids in the 10% LBP, 20% LBP, 10% KP, 10% LBP-10% HRP, and 10% KP-10% LBP-10% HRP groups were formic acid, citric acid, and fumaric acid. However, the main organic acids in the 20% KP, 30% KP, and control groups were formic acid, malic acid, and citric acid. Additionally, butyric acid was identified as one of the major organic acids in the 30% LBP group (Table 7). However, a previous study identified succinic acid as the main organic acid in shiitake mushrooms at different growth stages (75). Yang et al. reported that citric acid was the main organic acid in shiitake mushrooms, with a content of 24.45 mg/g (76). Gao et al. cultivated shiitake mushrooms using straw and found that malic acid was the main organic acid (12). Our results suggest that agro-forest waste used as a substitute for sawdust has an impact on the organic acid compounds present in cultivated shiitake mushrooms.

### 4.3 The acquisition of the optimal KP/HRP/LBP substrate formulation

Two exploratory data analysis methods, including principal component analysis (PCA) and membership function methods, were used to comprehensively assess the similarities and differences in the nutritional profiles of shiitake mushrooms grown on different substrates. The data presented in Tables 8, 9 demonstrate that the nutritional value of shiitake mushrooms is attributed to macromolecular nutrients, as well as volatile and non-volatile flavor components and other small molecules. Given this function and the vast amount of data generated, the statistical analysis method was employed after standardizing the data to explore and reveal the hidden relationships between the samples (77). Through factor transformation, PC1 was identified as primarily related to amino acid content; thus, it has also been referred to as the amino acid high-sensing factor. PC2 was mainly associated with soluble sugar content and can be designated as the sweetness factor. However, PC3 was mainly associated with the organic acid content and can be termed the organic acid perception factor (Figure 2). The comprehensive evaluation of factors and their rankings was ultimately derived through regression estimation. The results demonstrated that the treatment groups of 20%KP, 10%LBP, and 10%KP-10%LBP-10%HRP ranked among the top three. The amino acid perception factor (PC1) was dominant, while the non-volatile taste perception characteristics (PC3) presented an unacceptable risk. This further underscores the importance of amino acids in enhancing the taste and flavor selectivity of shiitake mushrooms.

Generally speaking, the market price for each ton of OS is 700 RMB, translating to a sawdust cost of 0.546 RMB per stick for cultivating *L. edodes* with the CK formula. Typically, a greenhouse (667 m^2^) can accommodate up to 8,000 mushroom sticks, leading to a total sawdust cost of 4,368 RMB for the greenhouse. By substituting OS with agro-forest waste for mushroom cultivation, we can save nearly half the cost of OS. Specifically, the 10%KP-10%LBP-10%HRP formulation used in mushroom cultivation results in a significant cost reduction, with sawdust for this formulation priced at 2,688 RMB. Notably, this method results in a saving of 1,680 RMB per greenhouse compared to the control. Furthermore, using the 10%KP-10%LBP-10%HRP formula for cultivating *L. edodes* in a greenhouse requires 2,400 kg of agro-forest waste, decreasing environmental pollution from these forest wastes. At the same time, the 2,400 kg of agro-forest waste can yield 3,500 kg of fresh shiitake mushrooms, expected to generate 35,000 RMB in economic benefits (calculated with 44% biological efficiency). Therefore, utilizing forest waste for mushroom cultivation and converting waste into economic value not only reduces environmental impact but also enhances resource recycling. Additionally, mushroom cultivation can generate employment opportunities in rural areas, increase farmers’ income levels, improve living conditions, and promote local economic development.

## 5 Conclusion

An appropriate substrate formulation is indeed a prerequisite for achieving high yields of shiitake mushrooms. Various forestry waste branches have a significant impact on the growth of shiitake mushrooms. In areas planted with *Lycium*, *Caragana korshinsk*, and *Hippophae rhamnoides*, it is advisable to utilize a substrate matrix formed by mixing oak sawdust with waste forestry branch residues. This approach leverages locally available resources and can potentially provide a suitable environment for growing shiitake mushrooms. The optimal substrate formulation has been identified as containing 20% KP, 58% OS, 20% bran, 1% sucrose, and 1% light calcium carbonate. The second-best formulation consists of 10% LBP, 68% OS, 20% bran, 1% sucrose, and 1% light calcium carbonate. Furthermore, the substrate mixture comprising 10% KP, 10% LBP, 10% HRP, 48% OS, 20% bran, 1% sucrose, and 1% light calcium carbonate has demonstrated excellent performance regarding growth rate, yield, and nutritional quality of shiitake mushroom mycelium. This combination can offer a balanced mix of nutrients, proper moisture-holding capacity, and good aeration, which are all essential for the healthy development of mycelium and the subsequent fruiting of shiitake mushrooms. The findings of this study open up new possibilities for better utilizing these residues, which are often abundant in certain areas, enabling mushroom growers to potentially increase their productivity and improve the overall quality of the mushrooms they produce, thereby gaining a competitive edge in the market and realizing greater economic benefits. Further research into how to enhance the yield of shiitake mushrooms cultivated with waste, shorten the cultivation period, and conduct large-scale experiments across different regions and environments will be worthy pursuits based on existing research.

## Data Availability

The raw data supporting the conclusions of this article will be made available by the authors without undue reservation.

## Supplementary material

SUPPLEMENTARY FIGURE S1 Significant correlations analysis between cultivation substrates and quality indicators of *L. edodes*.
