# Supplementary figures and images for: Enhancing yield and quality: research and practice of agro-forest waste for Lentinus edodes (shiitake mushroom) cultivation
Source: Front Nutr. 2025 Mar 19;12:1538039. doi: 10.3389/fnut.2025.1538039 (PMC11961442; doi:10.3389/fnut.2025.1538039)

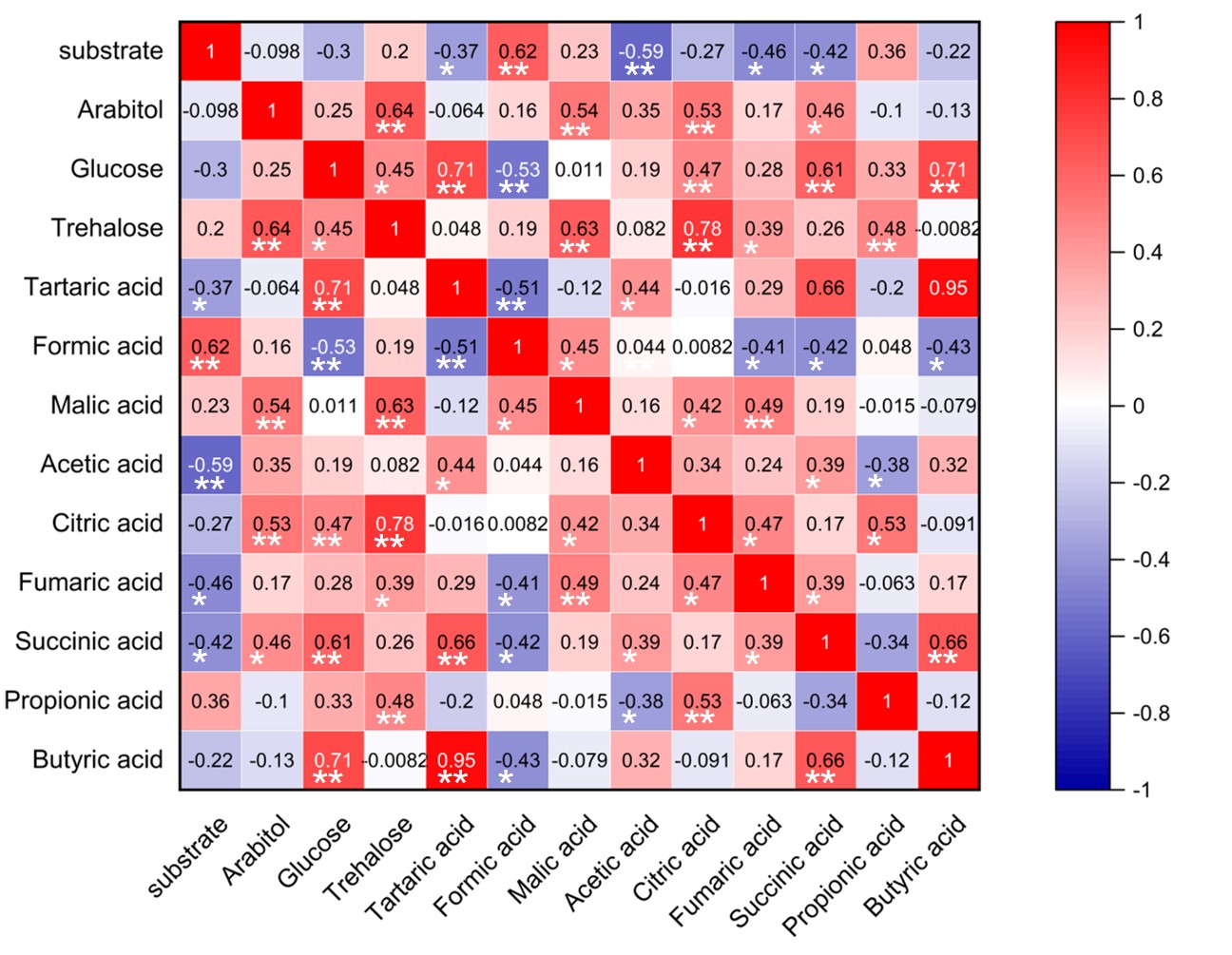

Supplement: SUPPLEMENTARY FIGURE S1 — Significant correlations analysis between cultivation substrates and quality indicators of L. edodes. [file Image_1.jpg]
